# Outcome of ductus arteriosus stenting including vertical tubular and convoluted tortuous ducts with emphasis on technical considerations

**DOI:** 10.1186/s43044-021-00210-4

**Published:** 2021-09-20

**Authors:** Saud Bahaidarah, Jameel Al-Ata, Naif Alkhushi, Ahmad Azhar, Zaher Zaher, Bayan Alnahdi, Mohamed Abdelsalam, Ahmed Elakaby, Ahmed Dohain, Gaser Abdelmohsen

**Affiliations:** 1 Pediatric Cardiology Division, Department of Pediatrics, King Abdulaziz University, P. O. Box 80215, Jeddah, 21589 Saudi Arabia; 2 Cardiology Department, Benha University, Benha, Egypt; 3 Paediatric Department, Al-Azhar University, Cairo, Egypt; 4 Pediatric Cardiology Division, Department of Pediatrics, Cairo University, Cairo, 11562 Egypt; 5 Paediatric Cardiology Division, Department of Paediatrics, Kasr Al Ainy School of Medicine, Cairo University, 99 El-Manial St., Cairo, 11451 Egypt

**Keywords:** Duct-dependent pulmonary, Single ventricle palliation, Patent ductus arteriosus, Stenting

## Abstract

**Background:**

Ductal stenting is the preferred method of securing adequate pulmonary blood flow in patients with duct-dependent pulmonary circulation. The main limitation in most centers is the difficult vertical tubular or convoluted ducts that represent real challenges to interventional pediatric cardiologists. We present our experience in patent ductus arteriosus (PDA) stenting with some technical tips to overcome difficulties, especially in stenting tortuous or long tubular ducts. This study was conducted on all patients with cyanotic congenital heart disease who underwent PDA stenting between January 2011 and December 2018.

**Results:**

We attempted to stent the PDA in 43 patients, with a success rate of 93% (40 patients) and only one procedural mortality. There was also one stent migration that needed to be treated with urgent surgery. Three-fourths of the patients had difficult ductal morphology and origin. One stent was used to cover the PDA in 27 patients (62.8%), two stents were used in 13 (30.2%), and three stents were used in 2 patients (4.6%). In-stent stenosis rate was 12.5% (5 patients) and the development of progressive left pulmonary artery stenosis was seen in two patients (5%). Pulmonary artery growth was adequate in all patients.

**Conclusions:**

PDA stenting is an effective method of palliation for patients with duct-dependent pulmonary circulation. It has low morbidity and mortality rates. Stenting difficult ducts have become more feasible with evolving materials and techniques.

**Supplementary Information:**

The online version contains supplementary material available at 10.1186/s43044-021-00210-4.

## Background

Stenting of the patent ductus arteriosus (PDA) is the primary treatment for patients with duct-dependent pulmonary circulation. It serves as a temporary bridge for later surgical repair [1]. It is considered a better alternative to conventional shunt surgery, owing to the latter’s high morbidity and mortality [2]. Two large studies found that PDA stenting was superior to Blalock–Taussig (BT) shunt placement, with fewer complications, shorter hospital stay, less use of diuretics, and better growth of pulmonary arteries [3, 4]. The outcome of ductal stenting has improved due to great advancements in coronary stent materials and design, and the improvement of guidewires and catheters, which in turn has led to greater success rates. However, the technical challenges of stenting long tubular or convoluted ducts are still a limiting factor in some centers [1]. Therefore, interventional pediatric cardiologists continue to modify their techniques of approaching, crossing, and stenting difficult ducts.

This study aimed to present our experience in patent ductus arteriosus (PDA) stenting with some technical tips to overcome difficulties, especially in stenting tortuous or long tubular ducts. Short- and mid-term outcomes of the procedure were also presented.

## Methods

This study included all patients with cyanotic congenital heart disease who underwent cardiac catheterization to stent the PDA between January 2011 and December 2018.

### Pre-procedure assessment

A detailed echocardiography was performed with special emphasis on the origin, morphology, and size of the PDA. Since most of our patients were neonates with a single source of pulmonary blood flow (PDA), intravenous prostaglandin administration was adjusted to the lowest possible dose required to maintain oxygen saturation at around 75%. We did not stop the prostaglandin except when the duct was approached, crossed with the wire (and occasionally with the stent), and angiography was performed, to accurately assess the morphology and size of the narrowest diameter of the PDA and to avoid any spasm while crossing.

### Procedure

The procedure was usually performed under general anesthesia, particularly in newborns. However, deep conscious sedation was occasionally used in older infants. Using the Seldinger technique, the femoral artery and vein were both accessed with a 4F sheath and a 5F sheath, respectively. Our approach to ductal stenting depended on ductal morphology, particularly, the origin of the ductus from the aorta, and the presence of a ventricular septal defect (VSD). The retrograde approach through the femoral artery was used in most patients, while the antegrade approach through the femoral vein was used in a few patients with tetralogy of Fallot/pulmonary atresia (TOF/PA), especially if the PDA originated from the undersurface of a right aortic arch. In those patients, the PDA was crossed either from the left side of the heart through a patent foramen ovale (PFO) or from the right side through a VSD. Both techniques are risky due to the possibility of either mitral valve injury, or variable degrees of heart block and arrhythmias while going through the VSD, especially in patients with congenitally corrected transposition of the great arteries (CCTGA).

Angiography was usually performed with a Judkins’ right (JR4) or pigtail catheter to determine the morphology, length, and diameter of the PDA, as well as the confluence of the pulmonary arteries (PAs) and the presence of stenosis. Ducts were classified into 4 groups according to their origin: arising from the proximal descending aorta (group A); arising from the undersurface of the proximal arch, i.e., vertical (group B); intermediate origin (group C); and arising from the subclavian or brachiocephalic artery (group D) [1].

In PDAs originating from the aortic arch undersurface, which represented most patients, we cut the pigtail catheter tip to form an inverted J to engage the ductal ampulla. However, we have recently adopted the use of the pigtail catheter without cutting the tip, as PDA spasm is more likely to occur due to ductal intimal injury by the sharp edge. Therefore, the new technique adopted involved placing the 4F Fep Pigtail® Merit Medical over the wire, allowing the leading part of the wire to straighten (open) the pigtail’s rounded distal end. Once at the arch, we withdrew the leading wire segment slowly back into the pigtail catheter and then pulled back the catheter slowly to drop into the PDA orifice with its normal soft rounded tip without causing ductal injury. Lately, the left internal mammary artery (LIMA) 4F catheter, which has a stiffer tip than the pigtail, had also been used to carefully engage the ampulla of the PDA. When the PDA arose from the proximal descending aorta or the arch branches, a JR4 catheter was maneuvered to engage the ampulla.

In the antegrade approach through the VSD to the aortic arch, a 5F Judkins’ right guiding catheter was preferred in accessing the PDA, as it provided good support when placing the stent and confirming its position through angiography using a Y-connector.

Once the catheter had been directed toward the PDA ampulla, multiple coronary wires (without any preference) were used to cross and were deployed into the PAs. Attempts resulting in straightening of the duct and ductal shape change with crossed wires mandated a repeat angiography, and the length of the PDA was re-measured to obtain the final stent length needed.

In the retrograde approach, the coronary stent was manipulated over the wire from the femoral artery access point up to the PDA without using long sheaths; based on fixed and stable anatomical and radiological reference points that have been predetermined before deployment. These points included the vertebral body and the intervertebral disk borders, the tracheal air column, the clavicle, and the tip of the endotracheal tube.

The type of stent used depended upon its availability. The stent needed to cover the entire ductal length from the pulmonary to the aortic ends, adding an extra 2–3 mm as a safety margin to ensure total coverage. The diameter of the stent was selected based on the patient’s weight and PDA diameter. A single stent between 15 and 23 mm was likely to be deployed in most patients. A long stent was likely to cause difficulty in tracking the wire, especially in tortuous or vertical tubular ducts. Therefore, instead of using a long stent, a shorter stent was deployed in the distal ductus followed by a second overlapping stent for the proximal part.

The first stent covered the lower curve of the duct from the pulmonary end into its mid-portion, with the second overlapping up to the subclavian artery to ensure coverage of the entire ductal length. Occasionally, a third stent was needed to cover the PDA origin at the subclavian artery, if left uncovered by the second stent.

### Post-procedure

After the procedure, all patients were kept ventilated for at least 6 h and given Enoxaparin 1 mg/kg/dose twice daily for 24 h, in addition to oral aspirin 5 mg/kg/day on the same night. Most of the patients were kept nil per orem (NPO) for 24 h after the procedure, especially low weight neonates, and feeding was gradually introduced as tolerated.

### Follow-up

The patients were seen regularly in the outpatient clinic, with the assessment of oxygen saturation and echocardiography to assess stent patency and pulmonary artery anatomy. Cardiac catheterization and angiography were indicated in patients with decreased oxygen saturation. The interventions were dilation, re-stenting of the PDA stent or diagnostic pre-surgical assessment, either for further repair or palliative procedures.

Echocardiography was performed before the procedure for diagnosis, evaluation of PDA size, shape, and origin. It was also done after the procedure for evaluation of ductal stent patency and pulmonary arterial growth during the follow-up period.

## Statistical analysis

Statistical analysis was performed using SPSS Version 26 software (IBM SPSS Statistics for Windows, Version 26.0. Armonk, NY: IBM Corp). Data were expressed as median and range (minimum–maximum) for numerical data, number, and percentage for categorical data. Comparison before and after intervention for oxygen saturation was tested using the Wilcoxon test. *P* value < 0.05 was considered of statistical significance.

## Results

PDA stenting was attempted in 43 patients (Table 1). The median age was 18 days (range 1 day to 6 years, with 9 patients after the neonatal period). The median weight was 3 kg (range 2.1–13 kg). Five patients (11.6%) were preterm, and 18 patients (41.9%) were female. The majority had pulmonary atresia with VSD (TOF/PA) (27.9%, 12/43), while pulmonary atresia with the intact interventricular septum (PA/IVS) represented 20.9% (9/43). The remaining patients had single ventricle physiology with pulmonary atresia or severely limited pulmonary blood flow.

**Table 1:** Patients’ characteristics

Patients’ demographics	
Age (days), median (range)	18 (1–2190)
Weight (kg), median (range)	3.1 (2.1–13)
Male/female, n (%)	25 (58.1)/18 (41.9)
Full term/preterm n (%)	38 (88.4)/5(11.6)
Diagnosis	
Pulmonary atresia/ventricular septal defect, *n* (%)	12 (27.9)
Pulmonary atresia/intact interventricular septum, *n* (%)	9 (20.9)
Pulmonary atresia/tricuspid atresia, *n* (%)	6 (13.9)
Complex congenital heart disease, *n* (%)	6 (13.9)
TGA-VSD-pulmonary stenosis, *n* (%)	4 (9.3)
Pulmonary atresia with a complete atrioventricular septal defect, *n* (%)	3 (6.7)
Pulmonary atresia with Ebstein anomaly, *n* (%)	2 (4.6)
Critical pulmonary stenosis, *n* (%)	1 (2.3)
Angiography	
Aortic arch sidedness, *n* (%)	
Left	30 (69.8)
Right	13 (30.2)
PDA length/diameter	
Diameter (mm), median (range)	2.4 (0.8–3.9)
Length (mm), median (range)	17.3 (9–35)
PDA origin, *n* (%)	
Origin from the undersurface of the aortic arch	16 (37.2)
Origin from proximal descending aorta	14 (32.5)
Origin from intermediate position	3 (6.7)
Origin from the subclavian or brachiocephalic artery (3 patients had double PDAs)	10 (23.2)
PDA morphology, n (%)	
Tortuous	23 (53.5)
Long tubular	12 (27.9)
Conical	8 (18.6)

*PDA* patent ductus arteriosus, *VSD* ventricular septal defect, *TGA* transposition of great arteries

The retrograde approach was used in most patients (38/43, 88.4%), while the antegrade approach was used in five patients (11.6%). Nearly one-third of our patients had a right aortic arch (30.2%, 13/43). The median PDA diameter was 2.4 mm (range 0.8–3.9 mm) and the median PDA length was 17.3 mm (range 9–35 mm).

**Fig. 1:**
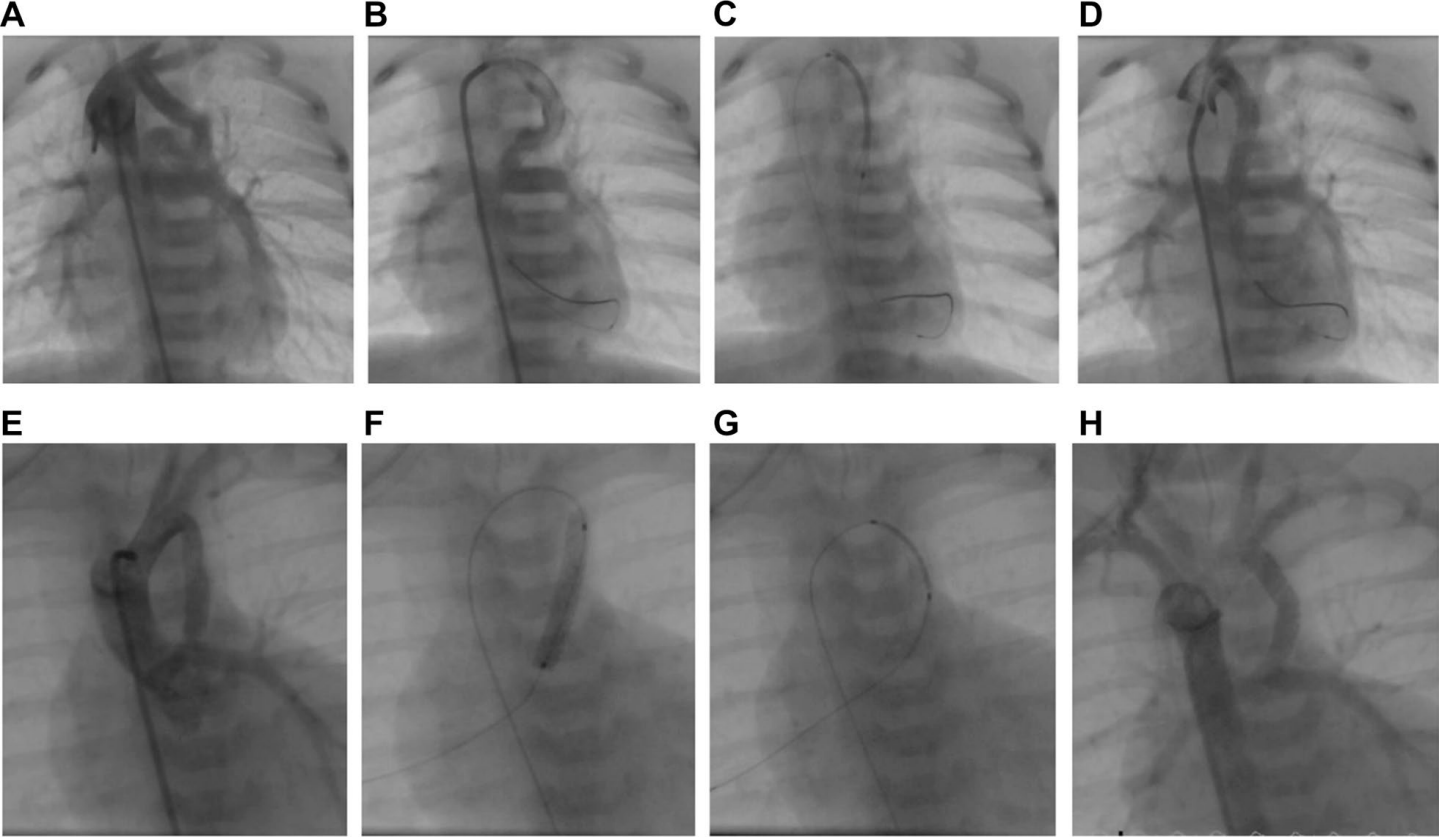
Stenting a long tubular patent ductus arteriosus (PDA) from the subclavian artery. Upper panel images **A**–**D** show one stent used to cover the PDA. Lower panel images **E**–**H** show the use of 2 stents

Ducts originating from the proximal descending aorta were found in 14 patients (32.5%), from the subclavian artery in 10 (23.2%), and the undersurface of the aortic arch in 37.2% (16/43). Three of our patients had double PDAs (all are successfully stented). The ducts were tortuous in 53.5% (23/43) and were vertical tubular ducts in 27% (12/43). PDAs originating from the left subclavian artery in the patients with a right aortic arch were noted to be C-shaped and were the longest ducts in our cohort (Fig. 1). These PDAs were usually 30–36 mm in length, and rarely less than 26 mm. For these ducts, using a very long stent would have probably lead to failure of the procedure or increase its risks, as the stent would straighten the C-shaped duct and stretch the PAs and the subclavian artery, increasing the likelihood of injury. Therefore, in such ducts, we used two or three stents (Fig. 1). Table 1 illustrates the demographic and angiographic characteristics of studied patients.

Successful PDA stenting was achieved in 40 patients with a success rate of 93%. In one patient, the stent migrated to the branch PAs, while the procedure failed in another with three overlapping stents. Both these patients were sent for surgical palliation. The third patient died before crossing the duct with the stent.

**Fig. 2:**
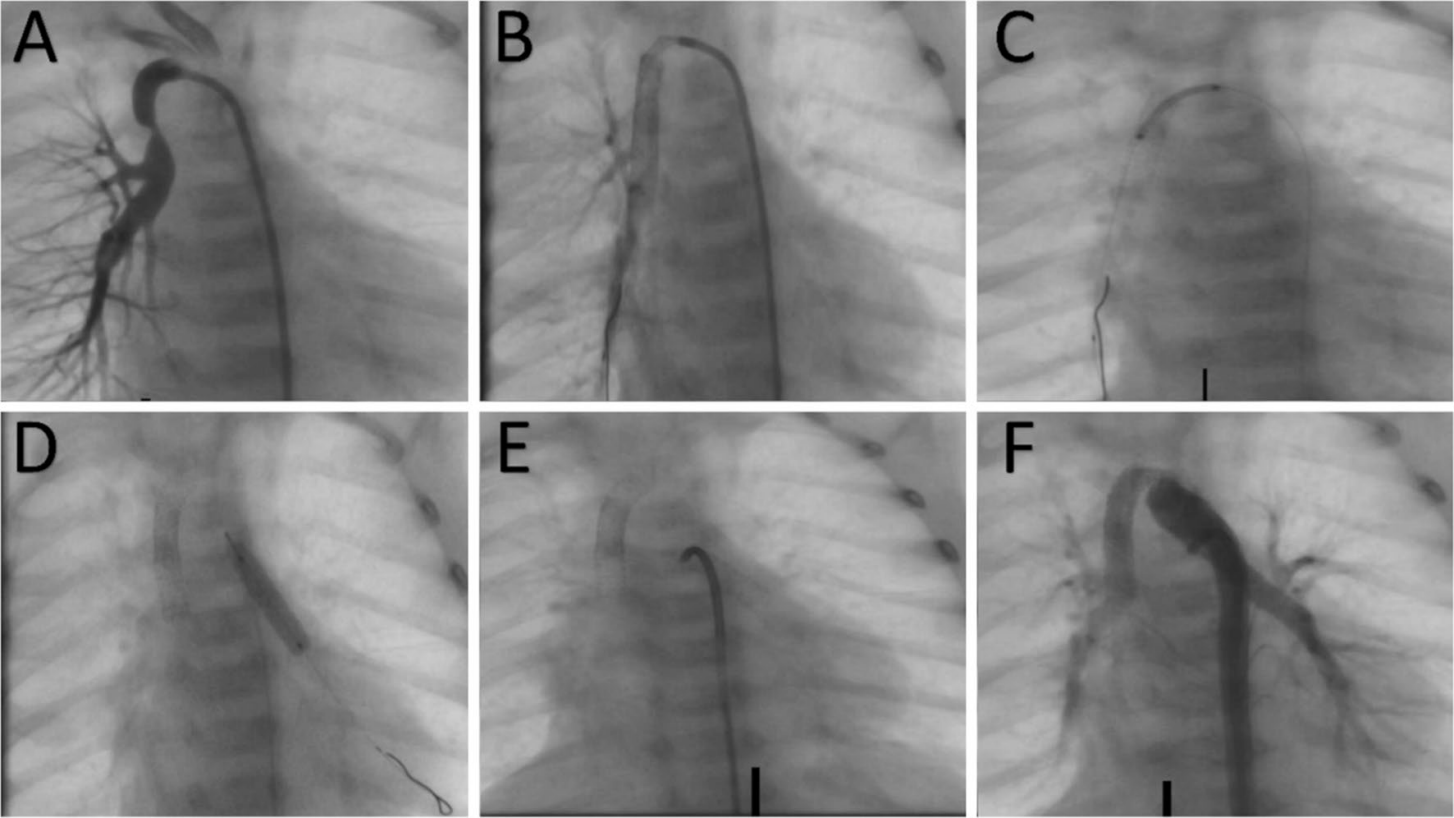
Stenting of double patent ductus arteriosus (PDA) in a patient with tetralogy of Fallot/pulmonary atresia and disconnected pulmonary arteries. The right pulmonary artery is supplied by a PDA from the subclavian artery that was stented with 2 stents (upper panel images, **A**–**C**). The left pulmonary artery is supplied by a PDA from the proximal descending aorta that was stented with one stent (lower panel images, **D**–**F**)

One stent was used to cover the entire length of the duct in 27 patients (62.8%), while two stents were used in 13 patients (30.2%). Only two patients (4.6%) used three stents: one of them had a double PDA (Fig. 2) and the other one had a right aortic arch and a very long C-shaped duct from the left subclavian artery. The median stent diameter was 3.5 mm, and the median stent length was 18 mm (Table 2).

**Table 2:** Cardiac catheterization data of studied patients

PDA access	
Retrograde, *n* (%)	38 (88.4)
Antegrade, *n* (%)	5 (11.6)
Properties of PDA stents	
Length (mm), median (range)	18 (12–30)
Diameter (mm), median (range)	3.5 (3–4.5)
Number of stents used	
One stent, *n* (%)	27 (62.8)
Two stents, *n* (%)	13 (30.2)
Three stents, *n* (%)	2 (4.7)
Fluoroscopy and IV contrast	
Fluoroscopy time (min), median (range)	13 (5–119)
Contrast volume (ml), median (range)	10.5 (2–21)
Procedure outcome	
Successful procedure, *n* (%)	40 (93)
Mortality, *n* (%)	1 (2.3)
Failure/stent migration, (sent for surgery), *n* (%)	2 (4.6)
In-hospital stay, days, median (range)	5 (1–278)
Follow-up (*n* = 40)	
In-hospital mortality, *n* (%)	6 (15)
Survival, *n* (%)	34 (85)

*PDA* patent ductus arteriosus, *IV* intravenous

Bare-metal stents (BMS) were preferred over drug-eluting stents (DES), as there still is not enough evidence regarding the off-label use of DES in such cases. Integrity bare-metal stents (Medtronic; Minneapolis, Minnesota) represented 37.9% of the stents used in our study. Multi-link vision stents (Abbott Vascular; Abbott Park, Illinois) was used in 41.2%, Challenger (Synexmed; Shenzhen, China) in 10.3%, Driver (Medtronic; Minneapolis, Minnesota) in 5.1%, Resolute Onyx (Medtronic; Minneapolis, Minnesota) in 3.4%, and PALMAZ BLUE™ (Cordis, Cardinal Health; Dublin, Ireland) in 1.7% of our patients. The median fluoroscopy time for the procedure was 13 min (range 5–119 min) and the median contrast volume was 10.5 ml.

### Immediate outcome

The median arterial oxygen saturation increased from 75 to 85% (*P* = 0.001).

There was one mortality due to the closure of the PDA. The patient died before the wire was passed or the stent was placed. The procedure was performed, as an emergency, on a critically ill baby with severe acidosis who was transferred from another hospital (Table 2).

Stent migration caused cardiac arrest in one patient who recovered after cardiopulmonary resuscitation. The stent was retrieved with concomitant BT shunt placement. Another patient was extubated accidentally and developed respiratory arrest. After difficult re-intubation and stabilization, the procedure was completed with no in-hospital sequelae. Sudden transient desaturation with no stent obstruction was observed in two patients (4.8%). One patient developed reperfusion injury due to excessive pulmonary over circulation.

### Hospital course

**Fig. 3:**
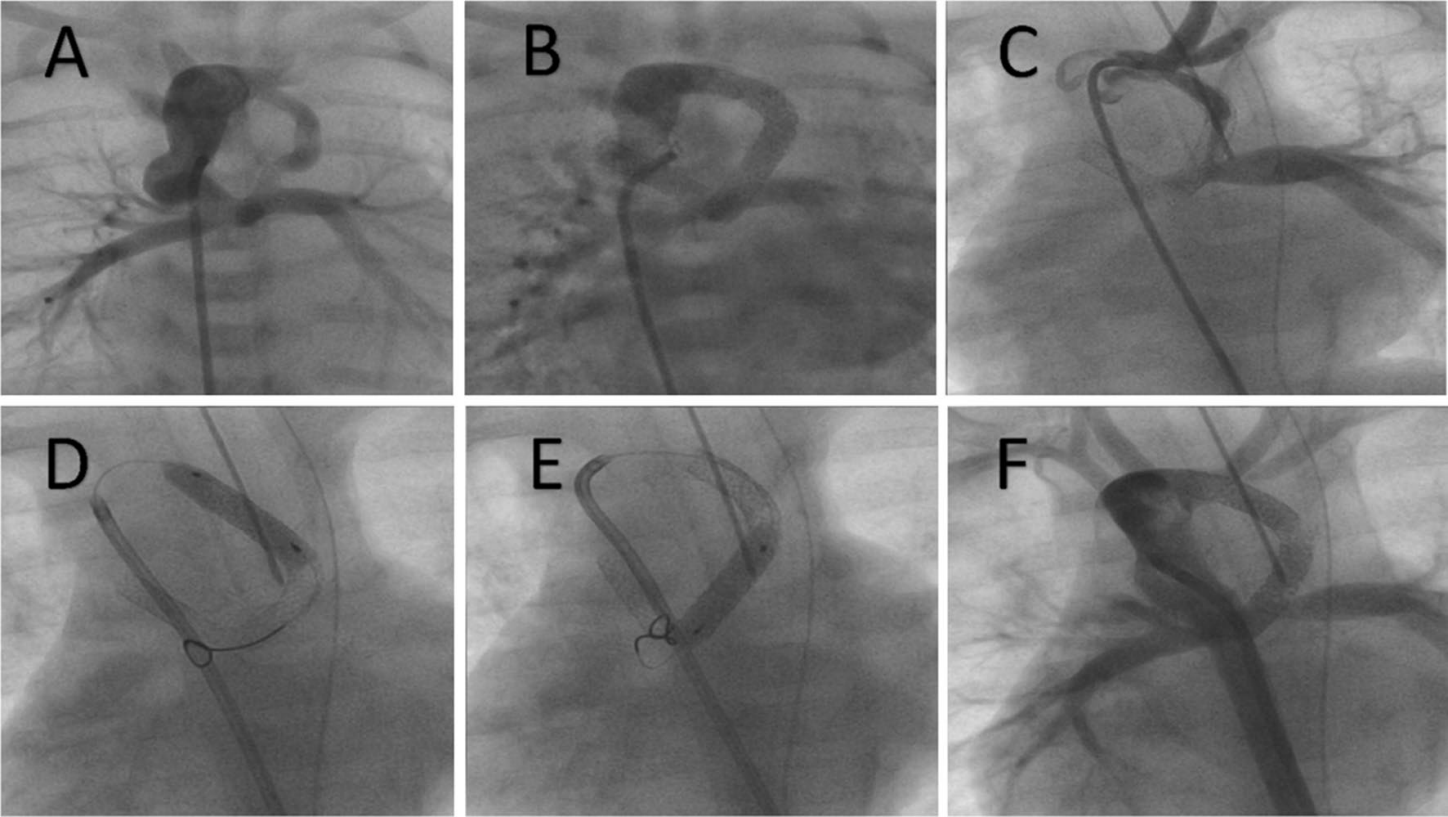
In-stent stenosis. This is a patient with tricuspid atresia/pulmonary atresia and double patent ductus arteriosus (PDA) (**A**), both stented due to severe confluence stenosis (**B**), presented a few months later with desaturation and in-stent stenosis (**C**). Re-stenting with 2 more stents (**D**, **E**) was done successfully (**F**)

The median duration of hospital stay was 5 days (range 1–278 days), with an in-hospital mortality rate of 15% (n = 6). One patient died due to acute stent thrombosis, two patients due to severe pneumonia, and three patients from fulminant sepsis. In-stent stenosis rate was 12.5% (5 patients) as seen in Fig. 3.

**Fig. 4:**
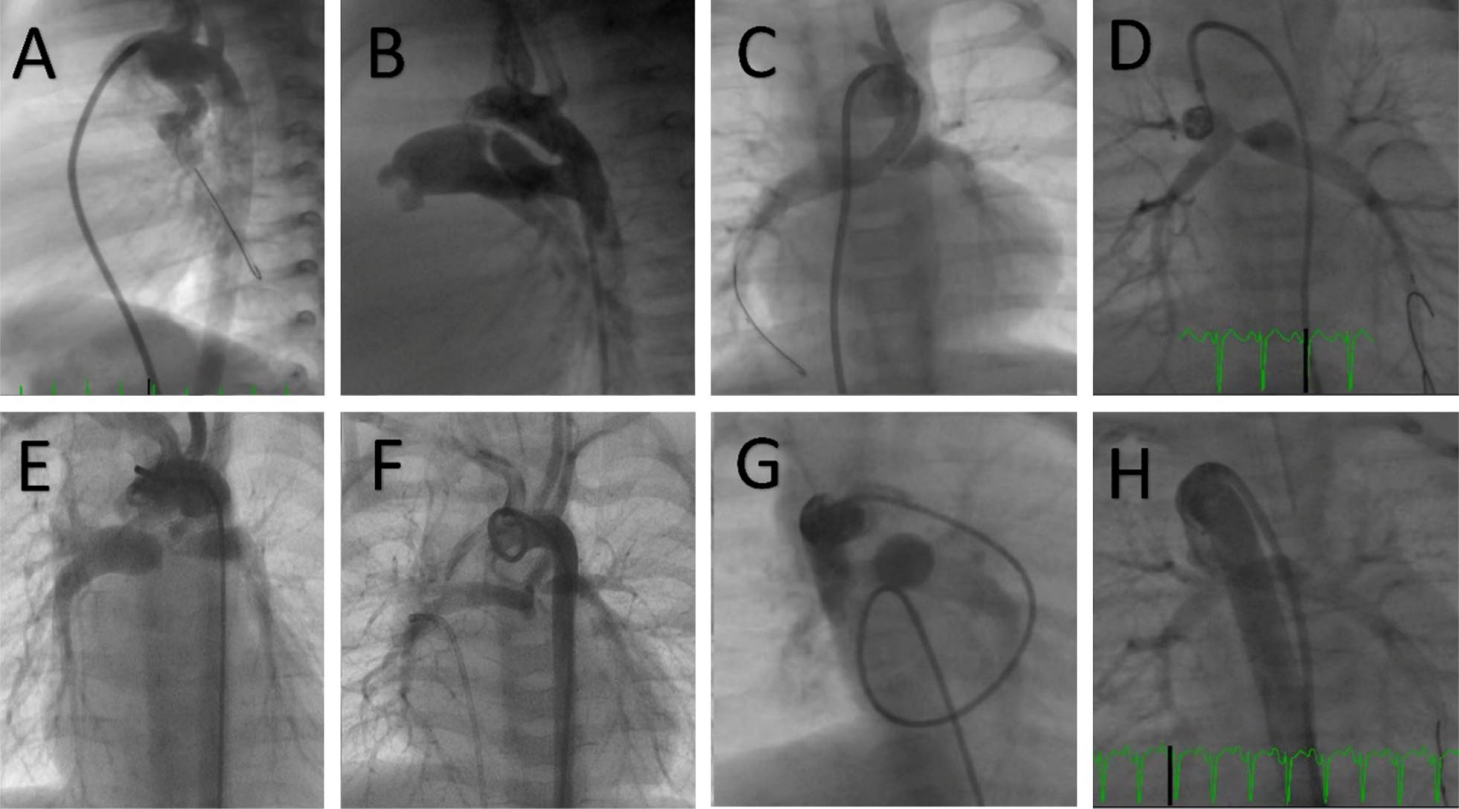
Left pulmonary artery (LPA) stenosis and growth of pulmonary arteries associated with ductal stenting. **A** The vertical duct from the undersurface of the aortic arch, **B** The regular conical duct from the proximal descending aorta. **C**, **D** Pre-existing LPA stenosis. On routine follow-up angiography, there was a development of stent stenosis and LPA stenosis (**E**, **F**). Follow-up angiography images show adequate growth of pulmonary arteries despite right pulmonary artery jailing (**G**, **H**)

Development of left pulmonary artery (LPA) stenosis after stenting was detected during cardiac catheterization in two patients, while pre-existing LPA stenosis did not show significant progression until the time of surgical repair in two TOF patients with preserved growth of the pulmonary arteries (Fig. 4). The rest of the patients showed adequate growth of the PAs by echocardiography or cardiac catheterization. Data for every patient were presented in a separate table/supplementary material (Additional file 1).

## Discussion

PDA stenting has emerged as an alternative to surgical shunt palliation. It has the advantages of being less invasive, thereby avoiding sternotomy and cardiopulmonary bypass, and potentially allowing for more symmetrical growth of the pulmonary arteries [5], aside from the shorter hospital stay compared to surgery [4]. However, PDA stenting in patients with duct-dependent pulmonary blood flow is still a Class IIb indication (level of evidence: C) according to the 2011 American Heart Association guidelines, and the presence of branch pulmonary artery stenosis is a Class III indication (level of evidence: C) [6].

Stenting long vertical or convoluted ducts always represents a challenge to interventional cardiologists and is a real limitation of PDA stenting in some centers [1]. Despite this, we were able to successfully palliate 40 patients with duct-dependent pulmonary blood flow (out of 43 attempts) via PDA stenting instead of creating surgical aortopulmonary shunts, irrespective of PDA origins and morphologies. We did not have any exclusion criteria concerning weights, ages, age of gestation (preterm), or anatomical diagnoses, unlike other reports that excluded tortuous, vertical, or very long ducts [7], as well as pulmonary artery stenosis [8].

Our practice is to keep the intravenous prostaglandin running until the duct is crossed with the wire. This technique may decrease the ductal spasm or enlarges the stenotic area while manipulating the wire in crossing trials, increasing the success rate of placing the wire deep in the lungs.

In our cohort, stenting was performed through femoral access without aggressive axillary or carotid arterial cannulation, or cutdown [9, 10], which we've observed to be the usual access point of engaging long and tortuous ducts with multiple curves and constrictions in other centers. Recent generations of softer and more floppy wires, flexible catheters with better torque, and stents with better profile and flexibility played major roles in safe, successful stenting through the femoral route.

Buys et al. [9] reported 80–100% procedural success while avoiding stenting tortuous and vertical ducts. Despite having a large proportion of tortuous and vertical ducts in our cohort (approximately 80%), our success rate was 93%, which is comparable to that of Alwi and other studies [7, 8, 11].

**Fig. 5:**
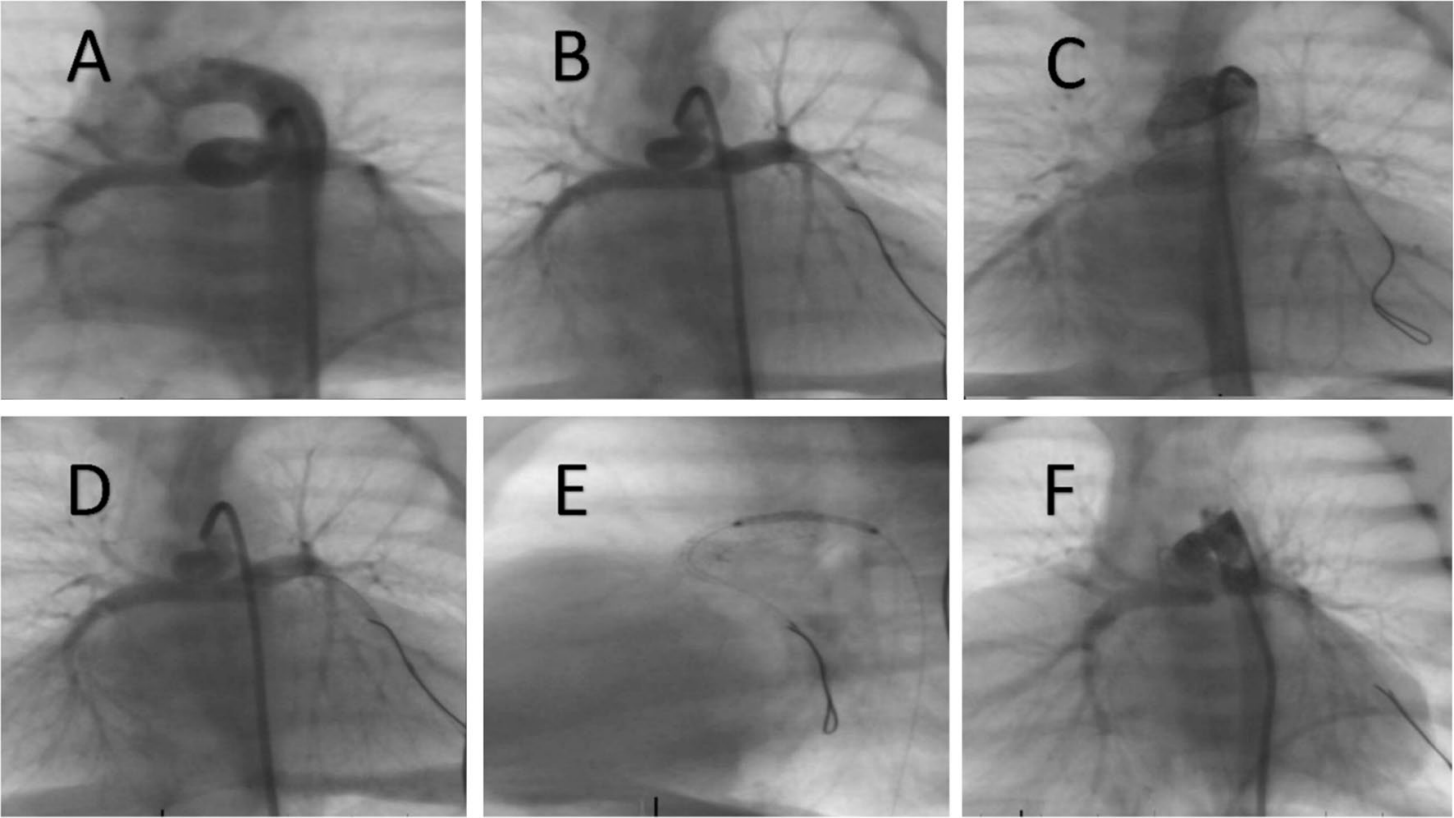
Stenting a convoluted duct. The upper panel images (**A**–**C**) show the tortuous duct with the wire taking the same complete circle of the duct. The wire was manipulated to straighten the duct (**D**). Two stents were used to cover the whole length of the duct (**E**–**F**)

**Fig. 6:**
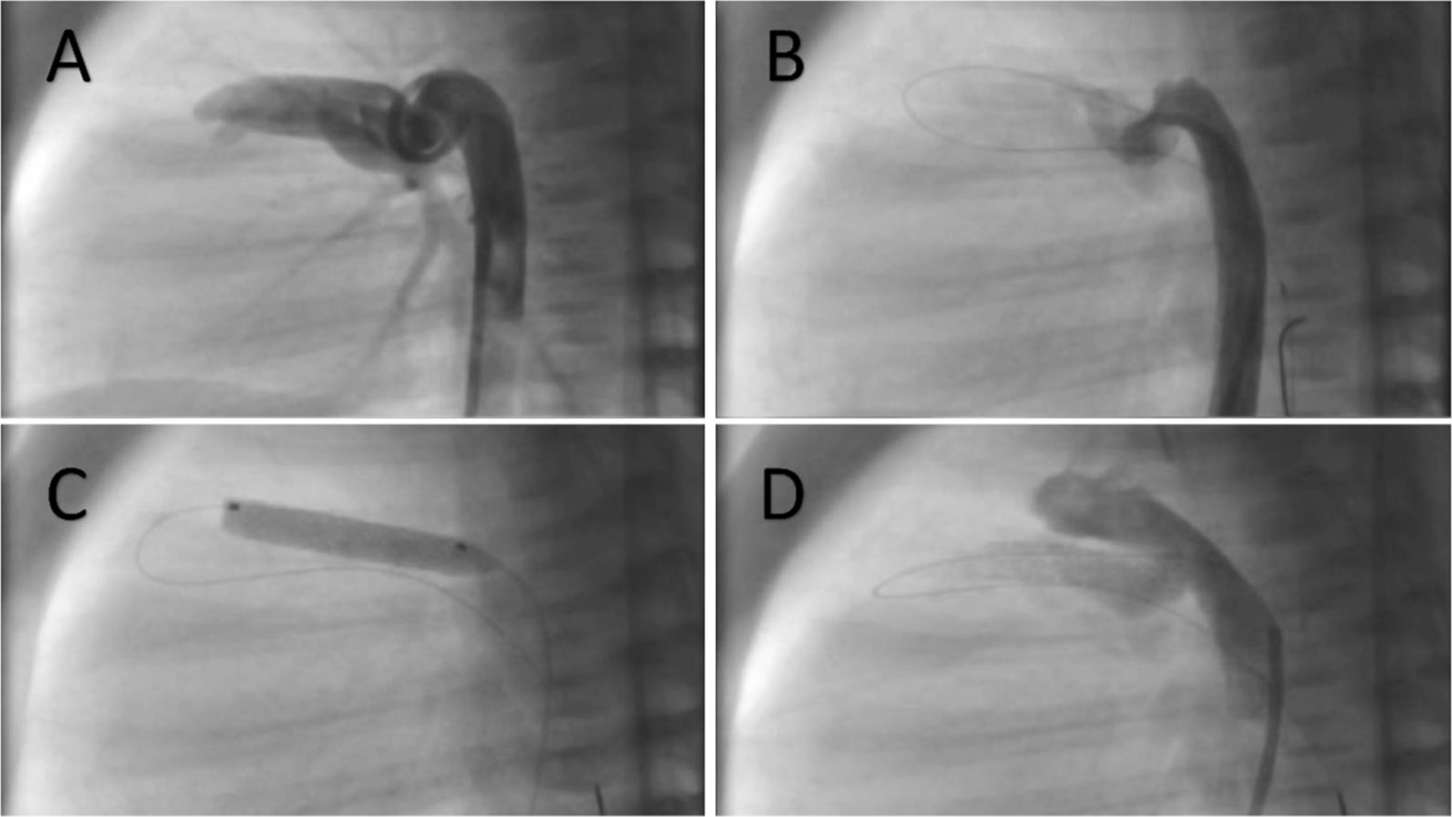
Stenting a tortuous duct. (**A**) Angiography showing a tortuous duct. The wire was pushed to the pulmonary artery stump then reflected to one of the pulmonary branches (**B**) to straighten the duct and stent it safely (**C**–**D**)

For convoluted tortuous ducts, it is important to straighten the duct (Fig. 5) by crossing the stump of the main pulmonary artery with the wire and then deflecting it back to the branch pulmonary artery, instead of crossing directly from the PDA to the branch PA (Fig. 6). The stents placed in a tortuous duct might change their orientation and position once the wire support is taken, which might result in uncovered ductal tissue constricting and causing desaturation. This can be avoided by straightening the duct with the wire and placing the stent based on the straightened length and adding a few millimeters to be within the aortic lumen, keeping in mind that the stent will cross the curves of the ductus and will not follow the initial tortuosity.

When it is difficult to cross the convoluted ducts with one long stent, stenting the distal pulmonary end first with a short stent, then overlapping with a second stent to cover the rest of the duct until the aortic end can be done. As studied and proven by Baho et al. [12], stent protrusion slightly into the pulmonary artery and sometimes the aorta causes no harm compared to leaving a part of the duct unstented.

Ductal length is of paramount importance, as underestimating it might leave an uncovered area that becomes stenotic while overestimating it may result in significant protrusion into the pulmonary artery or aorta.

It is very unlikely that a single stent of 15 mm or less in length will cover the entire PDA length. Most of these patients have underestimated PDA length due to tortuosity. Our single case of stent migration was in a patient stented with a short stent (< 15 mm).

We used more than one stent to adequately cover the PDA length in 15 patients (34.8%). Most of the trials used only one stent to cover the ductal length [7, 9, 11, 13]. Alwi et al. [8] used two stents in only one of 56 patients (1.7%). Two stents were used in a few of our patients because it is sometimes difficult to measure the length accurately with convoluted and angled ducts.

Three stents were used in two patients. One patient had disconnected PAs and a double PDA (Fig. 2); the right pulmonary artery (RPA) was supplied by a stenotic duct from the right subclavian artery, and the LPA was supplied by a regular duct from the aortic arch. Two stents were implanted in the ductus coming from the subclavian artery, and one stent was used for the other ductus. The other patient who required three stents had a right aortic arch with a long C-shaped ductus coming from the left subclavian artery.

Stents typically used for PDA stenting are a flexible and newer generation of coronary artery stents. About 80% of the stents used in our study were bare-metal stents. Drug-eluting stents have been reported to result in less in-stent restenosis than BMS when deployed in the PDA in an animal model [14]. Preliminary data on the use of DES and pharmacokinetics in neonates suggest significantly lower clearance of sirolimus in neonates and peak sirolimus levels being 20 times higher than in older children and adults [15]. Further studies are needed to assess the clinical relevance of this theoretical systemic immunosuppression.

Our reported periprocedural complication rates were low. Major complications included stent migration, cardiac arrest that required resuscitation, and subacute thrombosis. We did not have any case of permanent femoral vessel damage, contrary to a study by Glatz wherein this occurred in 9% of patients [3].

After the procedure, the neonates were kept on mechanical ventilation for 6–24 h to avoid acute reperfusion injury to the lungs. NPO was maintained for 24 h to avoid the possibility of gut ischemia after feeding due to the steal phenomenon from the PDA. Normal feeding was allowed after ensuring an absence of significant pulmonary over circulation.

There were a few in-hospital deaths (6 patients, 13.9%), mostly due to nosocomial sepsis and pneumonia. Most of the trials had a few deaths before hospital discharge, generally unrelated to stent implantation [7, 8, 10, 16–20].

In-stent stenosis has been reported to have a variable rate [8–10, 12] ranging from no restenosis at 6 months [21] up to 60%. This exceptionally high rate has been attributed to small stent sizes (3 and 3.5 mm) and incomplete coverage of the ducts [10]. Alwi had a stenosis rate of 16% [8]. In our group of patients, stent stenosis was detected in five patients (12.5%). The patients were successfully managed with catheter-directed re-interventions only. Two patients required repeat stenting for restenosis, and another two required balloon dilatations only. Repeated balloon dilatation or re-stenting has managed to maintain acceptable pulmonary blood flow to palliate inoperable patients for a period close to 4 years.

Pre-existing LPA stenosis is not uncommon, particularly in TOF/PA. Many investigators believe that PDA stenting tends to accelerate pre-existing branch PA stenosis [17, 22]. Alwi had seven patients with pre-existing mild LPA stenosis that progressed after stent implantation [8]. We only had two patients with pre-existing LPA stenosis, and we proceeded with stent implantation, with no progression in the degree of stenosis. Only two of our patients (nearly 5%) developed LPA stenosis, while the remaining patients had adequate growth of their PAs.

Partial or complete jailing of a branch PA was noted occasionally in our patients. It was noted in 22% of patients in one study [23]. However, in the long term, there was no significant difference in branch PA size and symmetry, regardless of whether or not a branch PA was jailed [22].

## Limitations of the study

The limitation of our study was largely the small number of cases, and this is explained by the rarity of such anomalies. It is also limited by its retrospective design.

## Conclusions

Patent ductus arteriosus stenting is a reliable first choice palliative strategy for patients with duct-dependent pulmonary blood flow. Difficult cases with long, vertical, or tortuous ducts can be approached safely and stented successfully. Using more than one stent is feasible and safe, to overcome difficulties and ensure complete coverage of the duct. Ductal patency can be maintained with adequate arterial oxygen saturation and balanced pulmonary artery growth until the patients can get their definitive repair or palliation.

## Supplementary Information

**Additional file 1**. Appendix to manuscript.

## Data Availability

All data were available at King Abdulaziz University Hospital.

## Abbreviations

BMS: Bare-metal stent
CCTGA: Congenitally corrected transposition of great arteries
DES: Drug-eluting stents
IVS: Intact ventricular septum
LIMA: Left internal mammary artery
LPA: Left pulmonary artery
NPO: Nil per orem
PA: Pulmonary atresia
PAs: Pulmonary arteries
PDA: Patent ductus arteriosus
RPA: Right pulmonary artery
TOF: Tetralogy of Fallot
VSD: Ventricular septal defect
