# Supplementary material for: Outcome of ductus arteriosus stenting including vertical tubular and convoluted tortuous ducts with emphasis on technical considerations
Source: Egypt Heart J. 2021 Sep 20;73:83. doi: 10.1186/s43044-021-00210-4 (PMC8452800; doi:10.1186/s43044-021-00210-4)
Supplement: Supplementary file 1 — Additional file 1. Appendix to manuscript. [file 43044_2021_210_MOESM1_ESM.docx]

| **Characteristics of patients who underwent PDA stents** | | | | | | | | | | |
| --- | --- | --- | --- | --- | --- | --- | --- | --- | --- | --- |
| Patient | Age (days) | Weight  (kg) | Diagnosis | Approach | PDA diameter (mm) | PDA length  (mm) | PDA type | No.of stents | Procedure outcome | notes |
| 1 | 7 | 3.2 | PA/IVS | Retrograde | 2.1 | 26.88 | Long tubular | 1 | Successful |  |
| 2 | 30 | 3.47 | TGA/VSD/PS | Retrograde | 2.3 | 12.86 | convoluted | 1 | Successful |  |
| 3 | 13 | 2.8 | PA/VSD | Retrograde | 1.8 | 19.5 | Long tubular | 2 | Successful |  |
| 4 | 90 | 2.6 | PA/IVS | Retrograde | 2 | 16.06 | Long tubular | 2 | Successful |  |
| 5 | 9 | 3.1 | PA/VSD | Retrograde | 2.6 | 18.4 | Long tubular | 2 | Successful |  |
| 6 | 23 | 2.6 | Critical PS | Retrograde | 2.4 | 26.38 | Long tubular | 1 | Successful |  |
| 7 | 2190 | 12.7 | PA/TA | Retrograde | 2.3 | 27.48 | regular | 2 | Successful |  |
| 8 | 3 | 3.2 | PA/AVSD | Antegrade | 2.2 | 20.8 | convoluted | 1 | Successful |  |
| 9 | 6 | 2.32 | PA/Ebstein anomaly | Retrograde | 3.2 | 22.18 | convoluted | 2 | Successful |  |
| 10 | 7 | 2.97 | Complex SV | Retrograde | 2 | 28.48 | convoluted | 2 | Successful | Double PDA |
| 11 | 30 | 3.24 | PA/IVS | Retrograde | 3 | 13.73 | regular | 2 | Successful |  |
| 12 | 23 | 3.1 | PA/VSD | Antegrade | 2.1 | 14.57 | convoluted | 1 | Successful | Double PDA |
| 13 | 28 | 3.7 | complex SV | Retrograde | 1.6 | 13.28 | convoluted | 1 | Successful |  |
| 14 | 24 | 2.9 | TGA/VSD/PS | Retrograde | 2.1 | 19.17 | convoluted | 1 | Successful |  |
| 15 | 19 | 2.5 | PA/TA | Retrograde | 2 | 19 | convoluted | 1 | Successful |  |
| 16 | 30 | 3.7 | PA/VSD | Retrograde | 2.7 | 13.25 | convoluted | 1 | Successful |  |
| 17 | 20 | 2.9 | PA/IVS | Retrograde | 3.3 | 20 | Long tubular | 2 | Successful |  |
| 18 | 9 | 3.2 | PA/IVS | Retrograde | 2.8 | 19.2 | convoluted |  | Successful |  |
| 19 | 30 | 3.75 | PA/VSD | Retrograde | 0.8 | 11.7 | convoluted | 1 | Successful |  |
| 20 | 6 | 2.4 | Complex SV | Retrograde | 2.4 | 14.63 | convoluted | 1 | Successful |  |
| 21 | 60 | 3.9 | PA/CAVSD | Retrograde | 2.6 | 20.89 | convoluted | 1 | Successful |  |
| 22 | 330 | 6.2 | Complex SV | Retrograde | 1.8 | 15.7 | convoluted | 1 | Successful |  |
| 23 | 365 | 9.5 | PA/TA | Retrograde | 1.7 | 14.3 | regular | 1 | Successful |  |
| 24 | 1 | 3.1 | PA/TA | Retrograde | 2.2 | 12.9 | Long tubular | 2 | Successful |  |
| 25 | 150 | 5 | PA/VSD | Antegrade | 3 | 17.6 | regular | 2 | Successful |  |
| 26 | 17 | 4.2 | PA/TA | Retrograde | 2 | 17.8 | convoluted | 1 | Successful |  |
| 27 | 9 | 3.3 | PA/Ebestein anomaly | Retrograde | 3.3 | 30.2 | Long tubular | 1 | Successful |  |
| 28 | 13 | 3.4 | Complex SV | Retrograde | 2.4 | 13.4 | convoluted | 1 | Failed/Urgent BT shunt |  |
| 29 | 24 | 2.8 | PA/VSD | Retrograde | 3.9 | 17 | convoluted | 1 | Successful |  |
| 30 | 60 | 2.7 | PA/VSD | Retrograde | 2.5 | 14 | convoluted | 2 | Successful |  |
| 31 | 13 | 2.32 | PA/TA | Retrograde | 1.9 | 12 | convoluted | 1 | Successful |  |
| 32 | 8 | 3.04 | PA/VSD | Antegrade | 2.2 | 15.2 | regular | 1 | Successful |  |
| 33 | 90 | 4.2 | PA/AVSD | Retrograde | 2.5 | 16 | Long tubular | 1 | Successful |  |
| 34 | 14 | 2.1 | PA/VSD | Retrograde | 1.4 | 35 | Long tubular | 3 | Failed/Urgent BT shunt |  |
| 35 | 28 | 13 | PA/VSD | Retrograde | 1.8 | 19.3 | Long tubular | 1 | Failed/ procedural mortality |  |
| 36 | 16 | 3.1 | TGA/VSD/PS | Retrograde | 1.5 | 15.5 | regular | 1 | Successful |  |
| 37 | 49 | 3.5 | TGA/VSD/PS | Retrograde | 1.1 | 17 | convoluted | 3 | Successful | Double PDA |
| 38 | 9 | 3.2 | PA/IVS | Retrograde | 3 | 18 | convoluted | 1 | Successful |  |
| 39 | 12 | 2.9 | Complex SV | Retrograde | 2.9 | 10 | convoluted | 1 | Successful |  |
| 40 | 6 | 2.8 | PA/IVS | Retrograde | 3.2 | 9 | regular | 1 | Successful |  |
| 41 | 9 | 2.7 | PA/VSD | Antegrade | 2.6 | 15 | Long tubular | 1 | Successful |  |
| 42 | 9 | 3 | PA/IVS | Retrograde | 2.8 | 24 | regular | 2 | Successful |  |
| 43 | 7 | 2.6 | PA/IVS | Retrograde | 2.3 | 18 | convoluted | 2 | Successful |  |
| ***Abbreviations:*** PA: pulmonary atresia, IVS: intact ventricular septum, PS: pulmonary stenosis, VSD: ventricular septal defect, AVSD: atrioventricular septal defect,  SV: single ventricle, TA: tricuspid atresia, TGA: transposition of great arteries, PDA: patent ductus arteriosus. | | | | | | | | | | |
